# Rosuvastatin Counteracts Vessel Arterialisation and Sinusoid Capillarisation, Reduces Tumour Growth, and Prolongs Survival in Murine Hepatocellular Carcinoma

**DOI:** 10.1155/2010/640797

**Published:** 2011-02-22

**Authors:** Annemilaï Tijeras-Raballand, Patricia Hainaud-Hakim, Jean-Olivier Contreres, Caroline Gest, Carole Le Henaff, Bernard I Levy, Marc Pocard, Claudine Soria, Evelyne Dupuy

**Affiliations:** ^1^Institut des Vaisseaux et du Sang, Hôpitlal Lariboisière, 2 Rue Ambroise Paré, 75010 Paris, France; ^2^“Angiogenèse et Recherche Translationnelle” INSERM U 965 “Equipe labellisée Ligue 2009”, Hôpital Lariboisière, 2 Rue Ambroise Paré, 75010 Paris, France; ^3^Université Paris 7 Denis Diderot, 10 Avenue de Verdun, 75010 Paris, France; ^4^Laboratoire M.E.R.C.I, Université de Rouen, 22 Boulevard Gambetta, 76183 Rouen Cedx, France; ^5^Hôpital Lariboisière, 2 Rue Ambroise Paré, 75010 Paris, France; ^6^Paris Cardiovascular Research Center, INSERM U970, Hôpital Européen Georges Pompidou, 20 Rue Leblanc, 75015 Paris, France; ^7^Hôpital Européen Georges Pompidou, 20 Rue Leblanc, 75015 Paris, France; ^8^Université Descartes-Paris 5, 12 Rue de l'École de Médecine, 75006 Paris, France

## Abstract

*Background and Aims*. An arterial blood supply and phenotypic changes of the sinusoids characterise the liver vasculature in human hepatocellular carcinoma (HCC). We investigated the effects of rosuvastatin on liver vessel anomalies, tumour growth and survival in HCC. *Methods*. We treated transgenic mice developing HCC, characterized by vessel anomalies similar to those of human HCC, with rosuvastatin. *Results*. In the rosuvastatin group, the survival time was longer (*P* < .001), and liver weight (*P* < .01) and nodule surface (*P* < .01) were reduced. Rosuvastatin decreased the number of smooth muscle actin-positive arteries (*P* < .05) and prevented the sinusoid anomalies, with decreased laminin expression (*P* < .001), activated hepatic stellate cells (*P* < .001), and active Notch4 expression. Furthermore, rosuvastatin inhibited endothelial cell but not tumour hepatocyte functions. *Conclusions*. Rosuvastatin reduced the vessel anomalies and tumour growth and prolonged survival in HCC. These results represent new mechanisms of the effects of statin on tumour angiogenesis and a potential target therapy in HCC.

## 1. Introduction

Hepatocellular carcinoma (HCC) is the fifth most common cancer in the world, with a poor prognosis. Vascular anomalies are the main characteristics of large and moderately or poorly differentiated HCC [1, 2]. Potential curative treatments are only available in highly selected patients. Sorafenib, an oral multikinase inhibitor targeting receptors involved in angiogenesis and tumour growth, is the only systemic treatment which has shown a modest, but significant, improvement in survival time in advanced HCC [3].

Among the various mechanisms involved in drug resistance, mitochondrial cholesterol could mediate resistance to chemotherapy in HCC [4]. Thus, statins, which inhibit 3-hydroxy 3-methylglutaryl coenzyme A (HMG-CoA) reductase, resulting in the decrease of mevalonate and cholesterol synthesis [5], could be interesting to use in combination with conventional therapies in HCC. However, clinical trials using statins in cardiovascular treatments have failed to provide conclusive results regarding their anticancer effects [6]. In contrast, a recent study demonstrated that statin treatment is associated with a significant reduction in the risk of HCC in diabetic patients [7]. Thus, statins might have a beneficial effect in HCC, likely in relation with their natural targeting towards hepatocytes [8]. Pravastatin treatment, alone or combined with chemoembolization, improves survival in patients with advanced HCC [9, 10]. 

The effects of statins on angiogenesis are disparate, depending on the drug concentrations and experimental conditions used [11–14]. Statins decrease the farnesylation and geranylgeranylation of small GTPase proteins involved in cellular survival, proliferation, and migration [15]. Ras and Rho, small GTP-binding proteins, are the main targets of statins [16]. By preventing RhoA localisation to the cell membrane, cerivastatin decreases endothelial cell (ECs) migration and reduces angiogenesis in matrigel and chorioallantoic membrane assays [17]. 

The concept described by Folkman four decades ago depicting that tumour neovascularisation is needed for tumour growth (the angiogenic switch) appears more complex than thought [18]. Some tumours such as HCC do not originate in avascular tissue [19, 20]. Severe anomalies of the liver vessels characterise human HCC [1, 2]. In contrast to the predominant portal venous blood supply and fenestrated sinusoids present in normal liver, an arterialisation is observed in HCC, characterised by an intense arterial blood supply with enlarged arteries and unpaired arteries, not associated with a bile duct. Furthermore, in tumour nodules, sinusoids are anarchic, fused, tortuous, and dilated. These sinusoids acquire a basal membrane rich in laminin and they are surrounded by activated hepatic stellate cells (HSCs) featuring sinusoid capillarisation. Activated HSCs express smooth muscle actin (SMA) and produce proangiogenic factor [1, 21] while normal sinusoids are surrounded by HSCs only expressing desmin [1, 2]. 

The aim of this study was to determine if statin, by preventing liver vessel anomalies, might delay tumour progression and prolong survival in HCC. For this purpose, we used transgenic mice developing HCC in a multistep angiogenic and tumour progression [22]. In this HCC model, we have previously described similar vessel anomalies to those of human HCC [23, 24] and have shown that PlGF blockade delays tumour growth and reduces arterialisation and sinusoid capillarisation [25].

The VEGF, Notch, and ephrin families play a key role in development of the vascular network and arterial/venous differentiation of blood vessels during embryogenesis and in the adult mouse [26, 27]. In the HCC model, Delta-like 4 ligand, the active form of the Notch4 receptor, and ephrin B2, expressed by sinusoidal endothelial cells (ECs), are gradually upregulated during HCC progression and facilitate the liver vessel anomalies [23]. 

Here, we have demonstrated a significantly longer survival rate, with reduced tumour growth, in rosuvastatin-treated HCC compared with untreated HCC mice. Rosuvastatin reduced the process of arterialisation of the liver vasculature resulting in smaller arteries and prevented sinusoid capillarisation, with a decrease in laminin and active Notch4 expression. Furthermore, the number of activated hepatite stellate cells surrounding the sinusoids was reduced under rosuvastatin treatment.

## 2. Materials and Methods

### 2.1. Reagents

The following antibodies were used for Western blots and immunofluorescence staining experiments: *A*, rabbit antismooth muscle actin (SMA) and rabbit anti-HIF-1*α* (Abcam, Cambridge, UK); rat antimouse CD31, (Pharmingen Becton Dickinson, Le Pont de Claix, France); rabbit anti-Notch4, recognizing the truncated intracellular domain of int3/Notch4 (Upstate, Euromedex, Mundolsheim, France and Abcam, Cambridge, UK); goat anti-*β*-actin (C11) (Santa Cruz Biotechnology, CA) and rabbit antilaminin (L-9393) (Sigma, Saint Louis MI, USA); and rabbit antidesmin (RB-9014) (Neomarker Inc. Fremont CA; USA). *B*, secondary antibodies for Western blots: donkey anti-goat IgG-peroxidase and donkey antirabbit IgG-peroxidase (Jackson Immuno Research Laboratory, West Grove, PA, USA); *C*, secondary antibodies for immunostaining: Alexa Fluor 488 and 555 goat antirabbit and Alexa Fluor 488 and 555 donkey anti-rat (Interchim, Asnières, France). 

### 2.2. Transgenic Mouse Model

Transgenic C57Bl6/ASV-B male mice developing HCC, in a multistep angiogenic and tumour progression from hyperplastic to the diffuse stage of HCC, have been described elsewhere [22]. Briefly, a precise targeting of the SV40 T early region expression in the liver of transgenic mice was achieved using 700 base pair of the antithrombin regulatory sequences to control oncogene expression. The development of hepatocarcinoma was restricted to male mice which were backcrossed with C57BL/6J mice. Mice were treated, or not (6 mice/group), with rosuvastatin given orally, daily (25 mg/kg), from the fourth week until death, for the survival study. The choice of rosuvastatin was determined by its hydrophilic properties, with minimum hepatic metabolism by cytochrome P450 systems [8]. For haematoxylin and eosin (HE) staining, immunofluorescence stainings, and Western blot studies, 4 supplementary mice/group were killed, at the diffuse stage (16 weeks) to obtain the liver samples. Animal procedures were conducted under anaesthesia, in accordance with French government policy (Services Vétérinaires de la Santé et de la Production Animale, Ministère de l'Agriculture).

### 2.3. Hepatocyte Isolation from HCC Livers and Cell Cultures

Hepatocytes were isolated from untreated HCC livers. The procedure was adapted according to Riccalton-Banks [28]. HCC mice (16 weeks) were anesthetised with Xylasine 20 *μ*g/g body weight (Bayer, Division santé animale, Puteaux, France) plus Ketamine 50 *μ*g/g body weight (Virbac, Carros, France) to perform the laparotomy and to expose the portal vein. The liver was perfused through the portal vein with a canula (22G, Vygon, Ecouen, France) connected to a peristaltic pump. The hepatic vein was split. The liver was perfused (flow rate 10 ml/min) with 250 ml perfusion buffer (0.2 M NaCl, 2.7 mM KCl, 0.7 mM Na_2_HPO_4_, 10 mM Hepes pH 7.65) at 37°C, followed by 100 ml perfusion buffer supplemented with 6.5 mM CaCl_2_ and 20 *μ*g/ml Liberase (Roche Diagnostics GmbH, Penberg, Germany) at 37°C at a flow rate of 5 ml/min. After perfusions, the liver was carefully excised and rinsed two times with 20 ml perfusion buffer. The liver capsule was peeled off and the liver was dissociated in 100 ml M199-10% FCS at 37°C, to dissociate the cells. The crude cell suspension was filtered through a 60-p.m mesh nylon screen (SCRYNEL NY60HC, VWR, Fontenay-sous-Bois, France) and sedimented for 30 minutes, at room temperature. The pellet, containing the hepatocyte fraction, was suspended in 100 ml M199-10% FCS and was washed four times (1000 rpm for 5 minutes). Hepatocytes (150 000 cells/cm^2^) were cultured in collagen-coated plates (0.15 mg/ml) (BD, Bedford, MA, USA) in DMEM-10% FCS plus 4.5 g/l glucose. After microscopic morphology evaluation, cells appeared well-differentiated, with a hepatocyte-like morphology. Tumour hepatocytes were expanded, frozen in 10% DMSO, and kept in liquid nitrogen. After thawing, cells were further passaged and used for the experiments.

Human endothelial cells (HUVECs) were isolated from the umbilical vein and cultured in EBM2-20% FCS plus 2 ng/ml FGF-2 as described [23].

### 2.4. Cell Proliferation and Matrigel Assays

For cell proliferation assays, HUVECs (15 000 cells/well), in EBM2-20% FCS, and tumour hepatocytes (50 000 cells/well), in DMEM-4.5 g/l glucose-10% FCS, were seeded in 6-well culture plates. After 72 hours, cells were cultivated with or without rosuvastatin at 10 *μ*M, 25 *μ*M, and 100 *μ*M for HUVECs and 100 *μ*M for hepatocytes. Cells were counted every 24 hours (Zeiss Axiovert 25, Carl Zeiss, Sartrouville, France). Results were the mean ± SD of three experiments done in quintuplicate.

For Matrigel assays, HUVECs (100 000 cells/well) were seeded on Matrigel gel (BD Biosciences, Le Pont-de Claix, France) in 12-well culture plates, in EBM2-20% FCS, in the presence or in the absence of rosuvastatin (100 *μ*M). Capillary-like structure formation was observed after 18 hours (Zeiss, Axio Observer Z.1). Images were obtained using a digital camera (Baumer TXD14, Radeberg, Germany). Three experiments were performed in duplicate. The number of sprouts was quantified with Histolab software (Microvision, Evry, France). Results were the mean ± SD of three experiments performed in duplicate. 

### 2.5. Invasion Assay

Tumour hepatocytes (75 000 cells/chamber), in serum-free medium, were seeded in the insert coated with matrigel (BD Biosciences), in the presence or absence of rosuvastatin (100 *μ*M). The lower chamber was filled with 750 *μ*l RPMI 1640 containing 10% FCS to induce chemotaxis. After 24 hours, the nonmigrated cells present in the upper chamber were gently scraped away, and adherent cells present on the lower surface of the insert were fixed with methanol, stained with 1% toluidine/1% borax solution, and counted with Mercator software (Explora Nova, La Rochelle, France). Results (number of invading cells) were the mean ± SEM of three experiments performed in duplicate.

### 2.6. Rho Activation Assay

Tumour hepatocytes or HUVECs were cultured for two days with or without rosuvastatin (100 *μ*M). The cells were then lysed and activated RhoA was measured by the G-Lisa kit, according to the manufacturer's instructions (Cytoskeleton Inc, Denver USA). Briefly, the active, GTP-bound RhoA, but not the inactive, GDP-bound form, in the biological sample binds to the rothekin-coated plate. Bound active RhoA is then detected by incubation with a specific anti-Rho primary antibody, followed by a secondary antibody conjugated to peroxidase. Results, expressed in absorbance (490 nm), are the mean ± SD of three experiments performed in duplicate. 

### 2.7. Protein Extraction and Western Blot

Protein extraction from liver biopsies (100–200 mg) was performed as previously described [23]. Protein concentrations were assessed using the BCA protein reagent assay kit (Pierce, Rockford, Ill, USA). Samples were boiled in 4X Laemmli buffer. Protein samples were resolved by electrophoresis through 4% to 12% gradient SDS-PAGE precast gels (NuPage, Invitrogen, Cergy Pontoise, France) under reducing conditions in MOPS buffer (NuPage, Invitrogen). After transfer, (iBlot Transfer device, Invitrogen), membranes were incubated overnight at 4°C with primary antibody against Notch4 (1/200), HIF-1*α* (1/1000), and *β*-actin (1/400), used as an internal loading control, then incubated with the appropriate secondary antibody (1/50 000). Antibody binding was revealed with the ECL system (Pierce, Rockford, Il USA). The results (mean ± SEM) were normalised to the *β*-actin levels and expressed as a percentage of the control. 

### 2.8. Immunofluorescence (IF) Staining

Liver sections were prepared as previously described [22]. The primary antibody was omitted, or incubated with an excess of blocking peptide as a negative control. Liver sections were incubated with primary antibody: monoclonal rat anti-CD31 (1/50), anti-laminin (1/100), anti-desmin (1/200), anti-Notch4 (1/100), and anti-SMA (1/50) and then with the appropriate secondary antibody (1/200). All immunostainings were analysed using a standard fluorescence microscope (Zeiss Axio Observer Z1, Microvision, Evry, France). 

Area staining and vessel quantifications were performed using Histolab software (Microvision) by a blinded investigator. Nodular size was determined by area measurement per optical field (3 optical fields per liver). CD31, laminin, desmin, and SMA sinusoidal expression were determined by area measurement per optical field (3-4 optical fields per liver) and SMA vessel density was obtained by vessel counts per section.

### 2.9. Statistical Analysis

The logrank test was performed for the survival time. Data were analysed statistically using the Student's *t*-test (Excel; Microsoft), where *P* < .05 was considered significant (*P < .05; **P < .01; ***P < .001).

## 3. Results

### 3.1. Rosuvastatin Prolongs Survival and Delays Tumour Growth in HCC Mice

**Figure 1:**
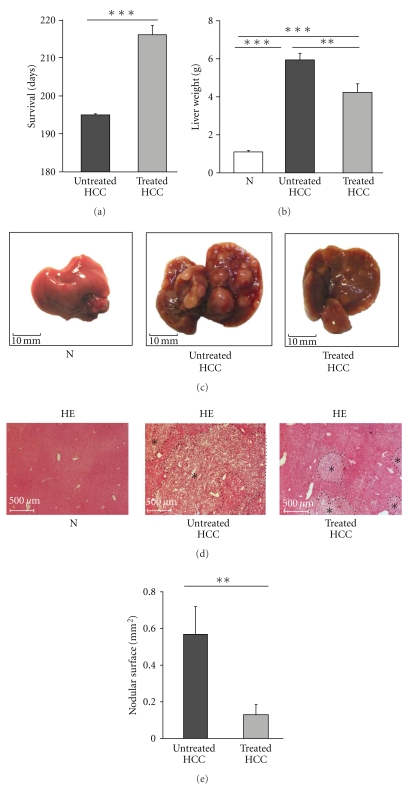
Rosuvastatin prolongs survival and delays tumour growth in HCC mice. (a) Survival rate of untreated mice (*n* = 6) and rosuvastatin-treated mice (*n* = 6). Data are mean ± SEM, ***P < .001. (b) Liver weight in normal mice (N) and in untreated and rosuvastatin-treated HCC mice (*n* = 4/group) at 16 weeks. Data are mean ± SEM, **P < .01, ***P < .001. (c) Representative images of normal livers (N) and untreated and rosuvastatin-treated HCC livers. (*n* = 4/group). Liver and nodule sizes were reduced under rosuvastatin treatment. (d) Representative images of haematoxylin-eosin (HE) staining in normal livers (N) and untreated and rosuvastatin-treated HCC livers (*n* = 4/group) at 16 weeks. Asterisks (∗) indicate nodules. (e) Quantification of the nodule surface in untreated and rosuvastatin-treated HCC livers (*n* = 4/group) at 16 weeks. Data are expressed as mean ± SEM (**P < .01).

Rosuvastatin-treated HCC mice showed a significantly longer survival time than the untreated HCC mice. The median survival time was 195 days (range: 193–198) in the untreated mouse group and 216 days (range: 214–220) in the rosuvastatin-treated HCC mouse group (*P* < .001) (Figure 1(a)). 

At 16 weeks, a significant increase in liver weight was noted in untreated HCC mice (5.87 ± 0.34 g) compared with normal wild-type mice (N) (1.1 ± 0.05 g) (*P* < .001) (Figure 1(b)). Rosuvastatin treatment significantly decreased the liver weight (4.18 ± 0.43 g, *P* < .01) (Figure 1(b)) and liver size (Figure 1(c)). Smaller nodules were present in HCC livers under rosuvastatin treatment (Figure 1(c)).

We then analysed the liver architecture by HE staining. At the diffuse stage of untreated HCC (16 weeks), large and fused tumour nodules were present within the liver tissue (Figure 1(d)). In contrast, at 16 weeks, smaller nodules were present within the liver tissue under rosuvastatin treatment (Figure 1(d)). Average nodule surface areas were significantly reduced in rosuvastatin-treated HCC (0.13 ± 0.054 mm^2^) compared with untreated HCC livers (0.57 ± 0.15 mm^2^; *P* < .01) (Figure 1(e)). Altogether, these results suggest that rosuvastatin delays HCC progression.

### 3.2. Rosuvastatin Prevents Sinusoidal Anomalies in HCC Livers

**Figure 2:**
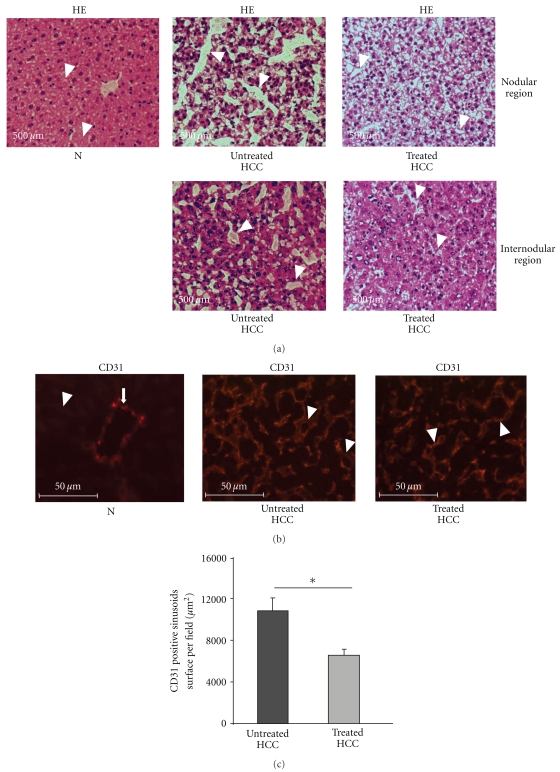
Rosuvastatin prevents sinusoid abnormalisation in HCC livers. (a) Representative images of haematoxylin-eosin (HE) staining in normal liver (N) and untreated and rosuvastatin-treated HCC livers (*n* = 4/group) at 16 weeks. Sinusoids are indicated by white arrowheads. Thin and regular sinusoids were observed in normal livers, whilst enlarged, tortuous, and fused sinusoids were present in untreated HCC livers. These sinusoidal anomalies were more marked within the nodular regions in untreated HCC livers. In rosuvastatin-treated HCC livers, these sinusoidal anomalies were less marked than those in untreated HCC livers. (b) Representative images of CD31 immunostaining in normal livers (N, *n* = 4) and in untreated and rosuvastatin-treated HCC livers (*n* = 4/group) at 16 weeks. CD31-stained endothelial cells lining vessels derived from the portal tract (VDPT) (white arrow) and endothelial cells lining the sinusoids (white arrowhead). (c) Quantification of the CD31-positive sinusoid surface area per field in untreated HCC (*n* = 4) and in rosuvastatin-treated HCC liver (*n* = 4) at 16 weeks. Data are mean ± SEM, *P < .05.

We analysed the sinusoids in normal livers and in the nodular and internodular regions in untreated and rosuvastatin-treated HCC livers (Figure 2(a)). As previously described [22], a thin, regular sinusoidal network was observed in normal livers, whilst fused, enlarged, and tortuous sinusoids were present in untreated HCC livers. These sinusoidal anomalies were more marked within the nodular than within the internodular regions (Figure 2(a)). The sinusoids were less extensively enlarged and chaotic in the nodular and internodular regions in treated compared with untreated HCC (Figure 2(a)).

We then carried out CD31 staining (Figure 2(b)). A very faint expression of CD31 in sinusoidal ECs with a regular sinusoidal network was present in normal livers (Figure 2(b)). In contrast, a strong expression of CD31 was observed in ECs lining the enlarged, tortuous, abnormal sinusoids in untreated HCC livers (Figure 2(b)). Interestingly, the sinusoids were less dilated and more regular, with a weaker CD31 expression in sinusoidal ECs under rosuvastatin treatment (Figure 2(b)). 

Rosuvastatin significantly reduced the sinusoid surface areas. The CD31-positive areas were 10863 ± 1762 *μ*m^2^ in untreated HCC livers versus 6589 ± 738 *μ*m^2^ in rosuvastatin-treated HCC (*P* < .05) (Figure 2(c)). 

### 3.3. Rosuvastatin Prevents Sinusoid Capillarisation in HCC Livers

In normal livers, the sinusoids are fenestrated vessels, without pericyte coverage, but surrounded by normal hepatic stellate cells (HSCs) expressing desmin. In HCC, the sinusoids develop into capillaries. They acquire a basal membrane rich in laminin and are surrounded by activated HSCs expressing SMA [1, 2, 25]. 

**Figure 3:**
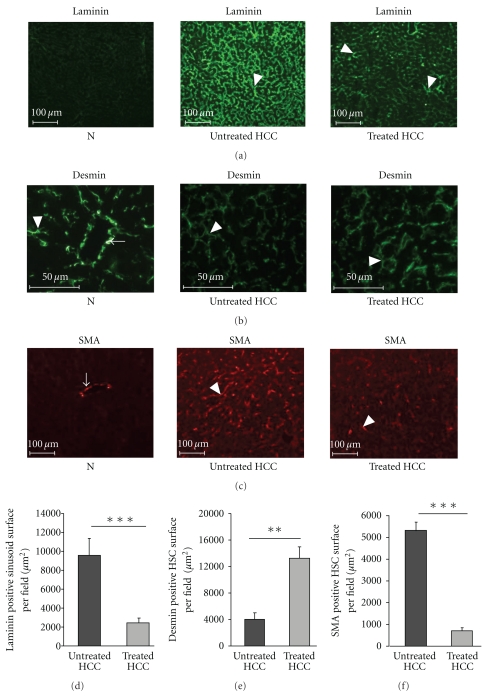
Rosuvastatin prevents sinusoid capillarisation in HCC livers. (a) Representative images of laminin immunostaining in normal livers (N, *n* = 4), untreated HCC, and rosuvastatin-treated HCC livers (*n* = 4/group) at 16 weeks. Laminin was expressed by the sinusoids (white arrowhead) only in HCC livers. A weaker expression of laminin was observed in treated-HCC livers compared with untreated HCC. (b) Representative images of desmin immunostaining in normal livers (N, *n* = 4), in untreated HCC, and in rosuvastatin-treated HCC livers (*n* = 4/group) at 16 weeks. Desmin was expressed in mural cells lining vessels derived from the portal tract (VDPT) in normal livers (white arrow). Desmin was expressed in nonactivated hepatic stellate cells (HSCs) surrounding the sinusoids in both livers (white arrowhead). (c) Representative images of SMA immunostaining in normal livers (N, *n* = 4), in untreated HCC, and in rosuvastatin-treated HCC livers (*n* = 4/group) at 16 weeks. SMA was expressed by smooth muscle cells lining arteries (white arrow). SMA was expressed in activated HSCs surrounding the sinusoids in HCC livers (white arrowhead). (d) Quantification of laminin-positive sinusoid surface per field, in untreated HCC and in rosuvastatin-treated HCC livers (*n* = 4: group) at 16 weeks. Data are mean ± SEM, ***P < .001. (e) Quantification of desmin-positive hepatic stellate cell (HSC) surface area per field in untreated HCC and in rosuvastatin-treated HCC livers (*n* = 4/group). Data are mean ± SEM, **P < .01. (f) Quantification of SMA-positive HSC surface area per field in untreated HCC and in rosuvastatin-treated livers (*n* = 4/group). Data are mean ± SEM, ***P < .001.

No laminin expression was depicted in normal livers whilst laminin was markedly expressed in untreated HCC livers (Figure 3(a)). In rosuvastatin-treated livers, a weaker laminin staining was observed (Figure 3(a)). Furthermore, the shape and size of the sinusoids were more regular under rosuvastatin treatment, confirming the HE and CD31 stainings (Figure 3(a)). We, then, compared the averaged surface of laminin-positive sinusoids in untreated and treated HCC livers. Rosuvastatin decreased laminin expression (laminin-positive areas: 2435 ± 562 *μ*m^2^/field in treated versus 9625 ± 1840 *μ*m^2^/field in untreated HCC livers (*P* < .001) (Figure 3(d)). 

We, then, performed CD31/desmin and CD31/SMA double-immunostainings, in order to analyse the HSCs phenotype. 

In normal livers, desmin stained mural cells in vessels derived from the portal tract and nonactivated HSCs (Figure 3(b), see Figure 1(a) in Supplementary Material available online at doi: 10.1155/2010/640797.), while SMA stained only arteries (Figure 3(c), Supplementary Figure 1(b)). The desmin-positive areas were higher in rosuvastatin-treated (13921 ± 1956 *μ*m^2^/field) than those in untreated HCC livers (4020 ± 1374 *μ*m^2^/field, *P* < .05) (Figure 3(e)). In normal livers, desmin-stained mural cells in vessels derived from the portal tract and SMA-stained smooth muscle cells in arteries (Supplementary Figures  1(a), 1(b). CD31-positive sinusoidal ECs were surrounded by non-activated HSCs expressing desmin in normal, untreated and rosuvastatin-treated HCC livers ((Supplementary Figure  1(a)) whilst they were surrounded by activated HSCs expressing SMA only in HCC livers (Supplementary Figure  1(b)). Interestingly, in rosuvastatin-treated HCC livers, CD31-positive sinusoidal ECs were more intensely surrounded by non-activated desmin-positive HSCs than in untreated HCC livers (Supplementary Figure  1(a)).

In normal livers, SMA stained smooth muscle cells in arteries (Figure 3(c)). Rosuvastatin markedly decreased the SMA-positive-activated HSCs compared with untreated HCC (Figures 3(c) and 3(f)). The average surface area of SMA-positive HSCs was 688 ± 147 *μ*m^2^/field in treated versus 5180 ± 389 *μ*m^2^/field in untreated HCC livers (*P* < .001) (Figure 3(f)). In treated HCC livers, CD31-positive sinusoidal ECs were less intensely surrounded by SMA-positive-activated HSCs than those in untreated HCC livers (Supplementary Figure 1(b)). Altogether, these data suggested that rosuvastatin impairs HSC activation and prevents sinusoid capillarisation in HCC. 

### 3.4. Rosuvastatin Prevents Arterialisation in HCC Livers

In healthy livers, the portal vein supplies the majority of hepatic blood flow. In contrast, HCC is characterised by an increase in the liver arterial blood supply, leading to an increase in the number of arteries [1, 2, 25].

**Figure 4:**
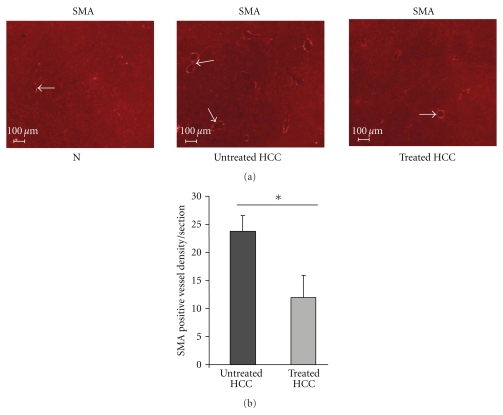
Rosuvastatin prevents arterialisation in HCC livers. (a) Representative images of SMA immunostaining in arteries in normal livers (N, *n* = 4), in untreated HCC and in rosuvastatin-treated HCC livers (*n* = 4/group) at 16 weeks. SMA stained smooth muscle cells lining arteries (white arrow). (b) Quantification of SMA-positive arteries per section in untreated HCC, and in rosuvastatin-treated livers (*n* = 4: group). Data are mean ± SEM, *P < .05.

We observed that rosuvastatin reduced the number of SMA-positive arteries in HCC compared with untreated HCC livers (12 ± 5.5/mm^2^/section for treated HCC versus 23.7 ± 4.3/mm^2^/section for untreated HCC, *P* < .05) (Figures 4(a) and 4(b)).

### 3.5. Rosuvastatin Reduces the Expression of Active Notch4 in Sinusoidal Endothelial Cells in HCC

**Figure 5:**
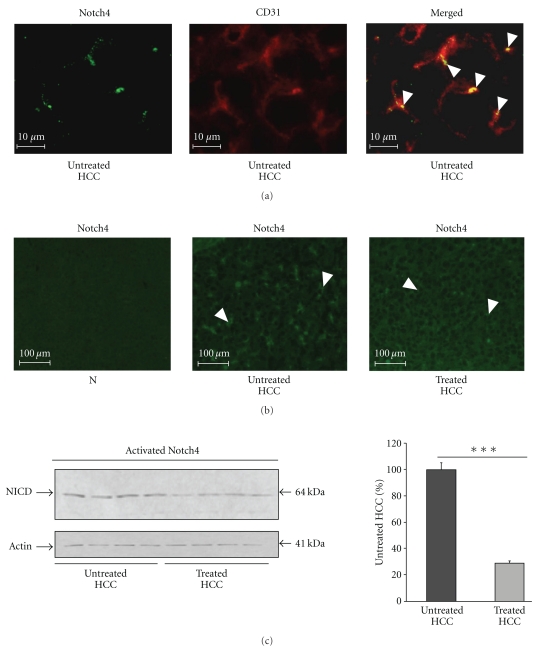
Rosuvastatin reduces the expression of active Notch4 in sinusoidal endothelial cells. (a) Representative images of CD31/Notch4 double immunostaining in untreated HCC livers at 16 weeks. We showed that Notch4 was expressed by CD31-positive endothelial cells lining the sinusoids (white arrowhead). (b) Representative images of Notch4 immunostaining in normal livers (N, *n* = 4), untreated HCC, and in rosuvastatin-treated HCC livers (*n* = 4/group) at 16 weeks (white arrowhead). (c) Western blot analysis of active Notch4 in untreated HCC and in rosuvastatin-treated HCC livers (*n* = 4/group) at 16 weeks. The results were normalised to the *β*-actin levels and expressed as a percentage of Notch4 levels in untreated livers. Rosuvastatin decreased significantly the levels of active Notch4 in HCC livers. Data are mean ± SEM, ***P < .001.

We have previously reported that tumour sinusoidal ECs change their phenotype and express the active form of Notch4, an arterial EC marker [23]. As previously reported, Notch4 was expressed by sinusoidal ECs in HCC livers (Figure 5(a)). Fewer sinusoids expressed Notch4 in treated compared with untreated HCC livers (Figure 5(b)). By western blot, we demonstrated that rosuvastatin significantly reduced the levels of active Notch4 by 71% (*P* < .001) in HCC livers (Figure 5(c)).

### 3.6. Rosuvastatin Reduces the Expression of HIF-1*α* in HCC Livers

**Figure 6:**
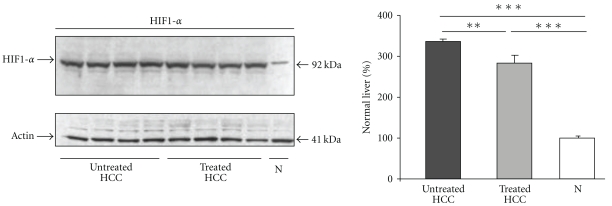
Rosuvastatin decreases HIF-1*α* levels in HCC livers. Representative image of western blot analysis of HIF-1*α* in normal livers (N), untreated HCC (*n* = 4), and in rosuvastatin-treated HCC livers (*n* = 4) at 16 weeks. The results were normalised to the *β*-actin levels and expressed as a percentage of HIF-1*α* levels in normal livers. Data are mean ± SEM. HIF-1*α* was significantly increased in HCC livers compared with normal livers, ***P < .001. Rosuvastatin decreased significantly HIF-1*α* levels in HCC livers, **P < .01.

We previously described that the HIF-1*α* messenger was increased in HCC livers [22]. Here, we showed, by western blot, that HIF-1*α* levels increased to 336 ± 7% in HCC livers compared with normal livers (Figure 6). Compared with untreated HCC, rosuvastatin significantly decreased HIF-1*α* levels to 284 ± 19% (*P* < .01) in HCC livers (Figure 6). 

### 3.7. Rosuvastatin Has No Effect on Tumour Hepatocyte Proliferation, Invasion, and Rho Activity

**Figure 7:**
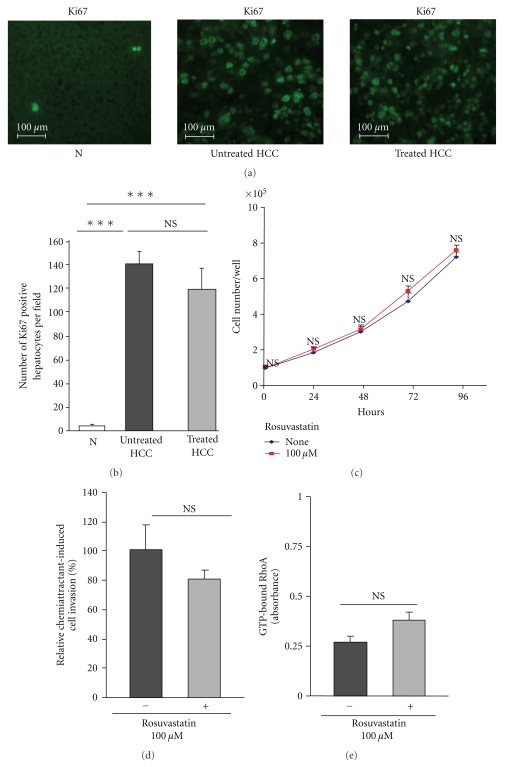
Effects of rosuvastatin on tumour hepatocyte functions. (a) Representative images of Ki67 immunostaining in normal livers (N, *n* = 4), untreated HCC, and in rosuvastatin-treated HCC livers (*n* = 4/group) at 16 weeks. (b) Quantification of Ki67-positive hepatocytes. Rosuvastatin did not affect the number of Ki67-proliferative hepatocytes in HCC. (c) Proliferation of cultured hepatocytes isolated from HCC livers with or without Rosuvastatin (100 *μ*M). Results, expressed as number of hepatocytes per well, are the mean ± S.D of three experiments performed in quintuplicate. (d) Tumour hepatocyte invasion with and without rosuvastatin treatment (100 *μ*M). Adherent tumour hepatocytes on the lower surface of the insert were counted with Mercator software. Results, expressed as a percentage of nontreated tumour hepatocytes, are the mean ± SD of three experiments performed in duplicate. (e) Rho activation assays in tumour hepatocytes with and without rosuvastatin treatment (100 *μ*M). Results, expressed as absorbance, are the mean ± SD of three experiments performed in duplicate, ***P < .001.

We first analysed if the delay in HCC progression was related to the effects of rosuvastatin on tumour hepatocytes. The numbers of Ki67 positive-cells/field did not differ significantly between treated (119 ± 59) and untreated HCC livers (140 ± 38) and were significantly higher than those of normal livers (4 ± 0.8, *P* < .001) (Figures 7(a) and 7(b)). 

Rosuvastatin (100 *μ*M) had no in vitro effect on tumour hepatocyte proliferation and invasion (Figures 7(c) and 7(d)). 

As Rho is a target of statins, we studied the in vitro activation of Rho in cultured tumour hepatocytes under rosuvastatin treatment. Rosuvastatin (100 *μ*M) did not modify the activation of Rho in tumour hepatocytes (Figure 7(e)).

Thus, our results suggest that Rosuvastatin does not act directly on tumour hepatocytes. 

### 3.8. Rosuvastatin Inhibits Proliferation, Differentiation, and Rho Activation of HUVECs

**Figure 8:**
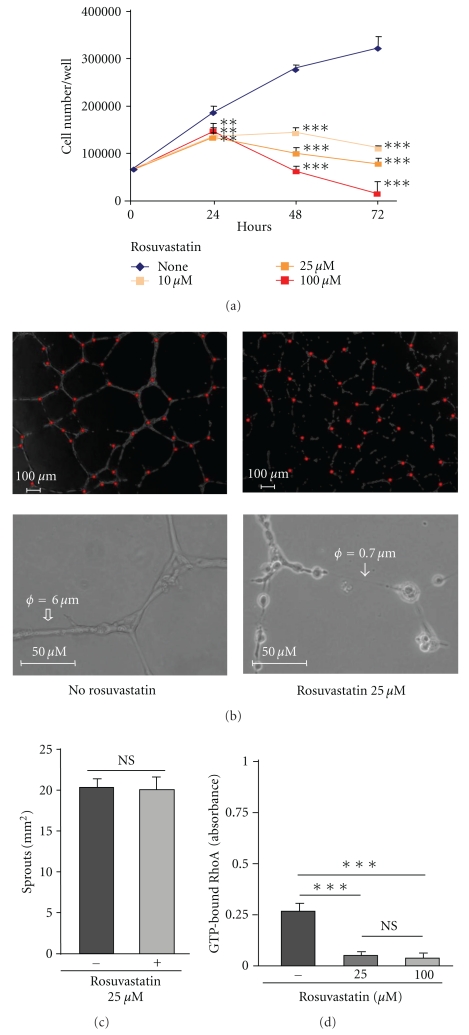
Effects of rosuvastatin on HUVEC functions. (a) Proliferation of HUVECs with or without rosuvastatin (0–100 *μ*M). Results, expressed as number of HUVECs per well, are the mean ± SD of three experiments performed in quintuplicate. (b) Effect of rosuvastatin (25 *μ*M) on the formation of capillary-like structures by HUVECs in Matrigel. (c) Quantifications of the number of sprouts/mm^2^ results were the mean ± SD of three experiments done in duplicate. (d) Rho activation assays in HUVECs with (25 *μ*M and 100 *μ*M) and without rosuvastatin treatment. Results, expressed as absorbance, are the mean ± SD of three experiments performed in duplicate, **P < .01 and ***P < .001.

We, then, analysed the in vitro effects of rosuvavastatin on HUVECs. The inhibition of HUVEC proliferation by rosuvastatin (10–100 *μ*M) was dose- and time-dependent (Figure 8(a)). After 24 hours, rosuvastatin inhibited HUVEC proliferation by approximately 25% for all concentrations. A dose-dependent effect of rosuvastatin was observed after 48 and 72 hours. After 48 hours, rosuvastatin significantly inhibited HUVEC proliferation by 49% (*P* < .001), 64% (*P* < .001), and 78% (*P* < .001) for 10, 25, and 100 *μ*M, respectively. After 72 hours, the inhibition reached 66% (*P* < .001), 76% (*P* < .001), and 95% (*P* < .001) for 10, 25, and 100 *μ*M, respectively (Figure 8(a)). 

In the absence of rosuvastatin, HUVECs differentiated into capillary-like structures with highly branched sprouting networks in Matrigel (Figure 8(b)). In the presence of rosuvastatin, HUVECS formed sprouts, and the numbers of sprouts did not differ significantly between untreated and rosuvastatin-treated HUVECs (Figure 8(c)). Under rosuvastatin treatment, HUVECs differentiated into cords without lumen rather than capillary-like structures (Figure 8(b)).

Similar results were obtained for proliferation and tube formation in Matrigel with human microvascular ECs (data not shown).

We then analysed the effect of rosuvastatin on Rho activation in HUVECS. Rosuvastatin inhibited Rho activation by 81% (*P* < .001) at 25 *μ*M and by 86% (*P* < .001) at 100 *μ*M (Figure 8(d)).

## 4. Discussion

Hypervascularity in human HCC is characterised by severe anomalies of the liver vasculature with an increased arterial blood supply, defined as arterialisation, and phenotypic modifications of the sinusoids, defined as capillarisation [1, 2]. In transgenic mice developing HCC in a multistep angiogenic and tumour progression [22], we have reported similar vessel anomalies to those in human HCC [23–25]. Thus, this model is appropriate to analyse the effects of rosuvastatin on liver vessel anomalies and tumour progression in HCC. As sorafenib is the only approved therapy for advanced HCC [3], we decided to analyse the effects of rosuvastatin at the diffuse stage (16 weeks) of HCC in this transgenic HCC model. 

Here, we have shown that rosuvastatin, in vivo, significantly reduces tumour growth, as scored by liver weight and nodule size, and significantly prolongs survival in transgenic mice developing HCC. HCC progression, resistance to chemotherapy, and intrahepatic recurrence have been reported to be modulated by Rho GTPase signals, the main targets of statins [29, 30]. In other rodent HCC models, statin reduced tumour progression, lung metastasis, and microvascular density. However, the effect of statins on survival was not established [31, 32]. The beneficial effect of statins in HCC may be related to their natural targeting towards hepatocytes [8]. Intriguingly, the number of proliferative hepatocytes did not differ between untreated and rosuvastatin-treated HCC livers. Furthermore, rosuvastatin did not inhibit, *in vitro*, the proliferation and invasion of tumour hepatocytes isolated from HCC livers and the activation of Rho in these hepatocytes. 

We, thus, focused on the in vivo effects of rosuvastatin on liver vasculature in HCC. In contrast to the predominant portal venous blood supply and the highly fenestrated sinusoids in normal livers, an intense arterial blood supply with an increase in unpaired arteries and a capillarisation of the sinusoid vessels characterises the HCC vascular network [1, 2]. We have previously shown, by ultrasonic measurements and microarteriographic studies, a predominant arterial blood supply with arterial-venous shunts in our model of HCC [24]. Furthermore, we also showed in this model that tumour sinusoids express laminin and that tumour sinusoidal ECs acquire an arterial phenotype [22, 23]. These similarities between the liver vascular network in human HCC and in our model prompted us to investigate whether rosuvastatin may counteract liver vessel anomalies in HCC. 

Here, we have shown that rosuvastatin significantly reduces the number of SMA-positive arteries, suggesting that rosuvastatin prevents arterialisation in our model of HCC. 

We also demonstrated that rosuvastatin counteracts the phenotypic changes of the sinusoids. Indeed, the sinusoids were less anarchic, tortuous, and dilated and had partially lost their basal membrane rich in laminin in rosuvastatin-treated HCC. Normal sinusoids, lacking pericytes, are surrounded by desmin-positive HSCs, while activated SMA-positive HSCs are present in HCC [1]. Similar anomalies were present in our HCC model. Indeed, desmin-positive HSCs were decreased, whilst activated SMA-positive HSCs were increased, in HCC compared with normal livers. Interestingly, rosuvastatin increased the number of nonactivated desmin-positive HSCs and decreased the number of activated SMA-positive HSCs in HCC.

Notch signalling regulates cell proliferation and differentiation and deregulated expression of Notch are described in HCC [33]. Overexpression of Notch1 inhibits the growth of HCC cells [34]. In contrast, downregulation of Notch1 signalling results in HCC cell growth inhibition and apoptosis [35]. 

Arterial-venous endothelial cell fate is regulated by the VEGF and Notch families [26, 36]. We have previously reported that the VEGF-A/Dll4/active Notch4/ephrine B2 cascade promotes liver vessel anomalies in HCC and that Notch4 is only expressed in tumour sinusoidal ECs [23]. Constitutive activation of Notch4 induces arteriovenous malformations in adult mice, notably in the liver [37]. Notch4 signalling promotes lung and brain arteriovenous malformations in mice [38, 39]. Since Notch4 signalling induces vessel anomalies, we analysed the liver expression of active Notch4 rather than Notch1 under rosuvastatin treatment. Here, we showed that rosuvastatin reduced the levels of the active form of Notch4, with fewer Notch4-positive sinusoids. Such a decrease in active Notch4 expression contributed, in part, to the beneficial effect of rosuvastatin on liver vessel anomalies in the HCC model.

Even if hypervascularity is a hallmark of HCC, liver vessel anomalies lead to impaired functional perfusion of HCC livers and facilitate hypoxia [1, 25]. Hypoxia induces the synthesis of angiogenic cytokines and stimulates HCC cell growth and resistance to therapy [40]. Moreover, activated HSCs secrete angiogenic factors and impair oxygen delivery [1, 41, 42]. In favour of this hypothesis, we have previously demonstrated an increase in HIF-1*α* messenger levels in the HCC model [22]. Here, we showed an increase in HIF-1*α* protein levels in untreated HCC compared with normal livers. Interestingly, we showed that HIF-1*α* was impaired under rosuvastatin treatment. Blockade of this cycle may improve functional liver vascularisation and the efficiency of therapies in HCC. 

To our knowledge, these effects of statins on the prevention of arterialisation and sinusoid capillarisation in HCC have not been previously reported.

Statins may promote or inhibit EC angiogenesis in vitro [11–14]. Here, we showed that rosuvastatin inhibits HUVECs proliferation, disorganizes the capillary network, but not the sprout number, in Matrigel, and decreases the activation of Rho.

Taken together, our data show that rosuvastatin prevents the capillarisation of sinusoids and the arterialisation of the liver vasculature, which may lead to reduced tumour growth and longer survival in HCC. Rosuvastatin inhibited ECs but not tumour hepatocyte functions, which might suggest that rosuvastatin targets endothelial cells rather than tumour hepatocytes. These results represent a new mechanism of the effects of statins on liver vessel normalisation in HCC and suggest a potential target to optimise therapies in advanced HCC. 

## Supplementary Material

In normal livers, desmin-stained mural cells in vessels derived from the portal tract and SMAstained smooth muscle cells in arteries (Supplementary Figure 1(a, b). CD31-positive sinusoidal ECs were surrounded by non-activated HSCs expressing desmin in normal, untreated and rosuvastatin-treated HCC livers ((Supplementary Figure 1(a)) whilst they were surrounded by activated HSCs expressing SMA only in HCC livers (Supplementary Figure 1(b)). Interestingly, in rosuvastatin-treated HCC livers, CD31-positive sinusoidal ECs were more intensely surrounded by non-activated desmin-positive HSCs than in untreated HCC livers (Supplementary Figure. 1(a)).
Representative images of negative controls. The primary antibody was omitted, or incubated with an excess of blocking peptide as a negative control (Supplementary Figure. 2).
