# Supplementary material for: Rosuvastatin Counteracts Vessel Arterialisation and Sinusoid Capillarisation, Reduces Tumour Growth, and Prolongs Survival in Murine Hepatocellular Carcinoma
Source: Gastroenterol Res Pract. 2011 Feb 22;2010:640797. doi: 10.1155/2010/640797 (PMC3082163; doi:10.1155/2010/640797)
Supplement: Supplementary file 1 — In normal livers, desmin-stained mural cells in vessels derived from the portal tract and SMAstained smooth muscle cells in arteries (Supplementary Figure 1(a, b). CD31-positive sinusoidal ECs were surrounded by non-activated HSCs expressing desmin in normal, untreated and rosuvastatin-treated HCC livers ((Supplementary Figure 1(a)) whilst they were surrounded by activated HSCs expressing SMA only in HCC livers (Supplementary Figure 1(b)). Interestingly, in rosuvastatin-treated HCC livers, CD31-positive sinusoidal ECs were more intensely surrounded by non-activated desmin-positive HSCs than in untreated HCC livers (Supplementary Figure. 1(a)). Representative images of negative controls. The primary antibody was omitted, or incubated with an excess of blocking peptide as a negative control (Supplementary Figure. 2). [file 640797.f1.pdf]

Tijeras-Raballand et al. Supplementary Figure 1

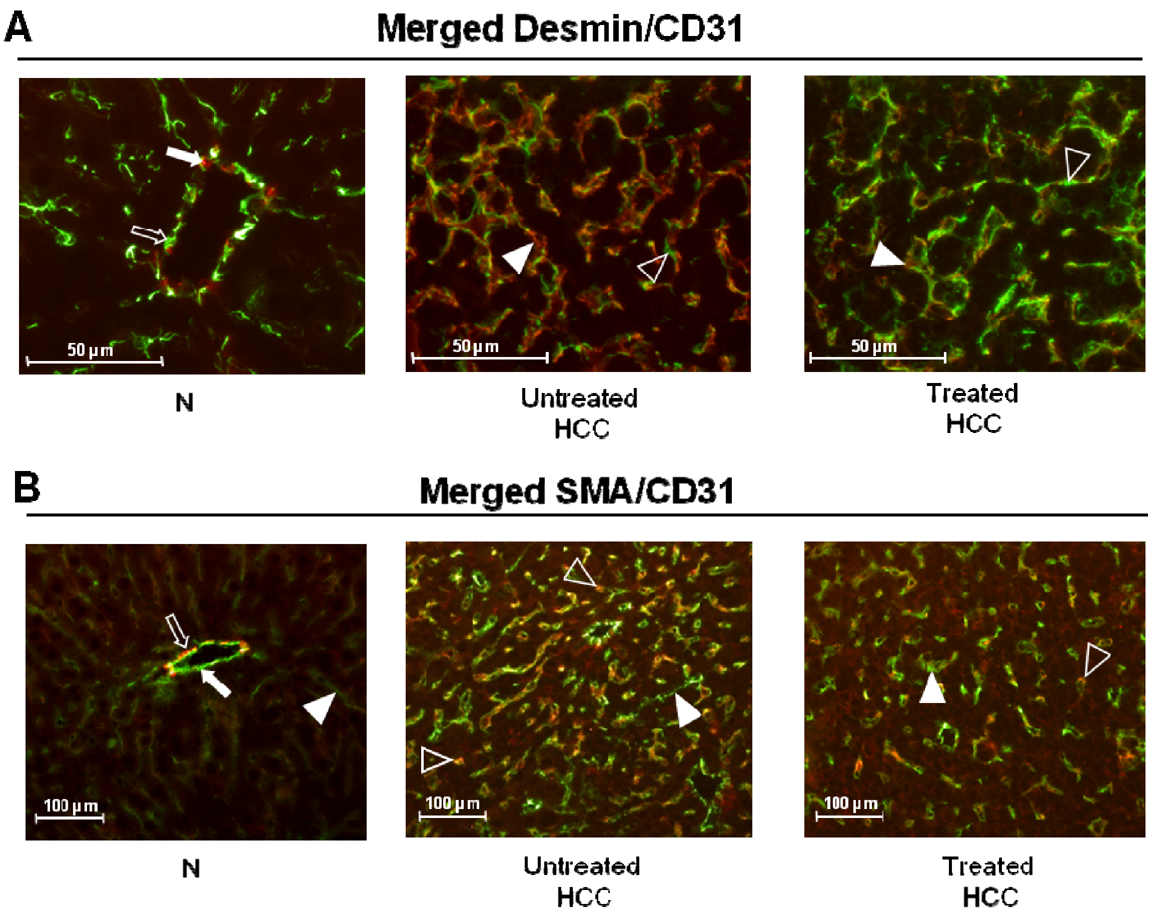

Supplementary Fig. 1

A, Representative images of Desmin (green)/CD31(red) double immunostaining in normal livers (n=4), untreated HCC and rosuvastatin-treated HCC livers (n=4/group). CD31-stained endothelial cells in VDPT (white arrow) and sinusoids (white arrowhead). Desmin-stained mural cells in vessels derived from the portal tract (empty arrow) and non-activated HSCs surrounding the sinusoid vessels (empty arrowhead). CD31-positive

sinusoidal endothelial cells were more surrounded by desmin-positive HSCs under rosuvastatin treatment in HCC livers.

B, Representative images of SMA (red)/CD31(green) double-immunostaining, in normal livers (n=4), untreated HCC and rosuvastatin-treated HCC livers (n=4/group). CD31-stained endothelial cells in VDPT (white arrow) and sinusoids (white arrowhead). SMA-stained smooth muscle cells in arteries (empty arrow) and activated HSCs (empty arrowhead) surrounding the sinusoids. CD31-positive sinusoidal endothelial cells were less surrounded by SMA-positive activated HSCs under rosuvastatin treatment in HCC livers.

Tijeras-Raballand et al. Supplementary Figure 2

Negative controls

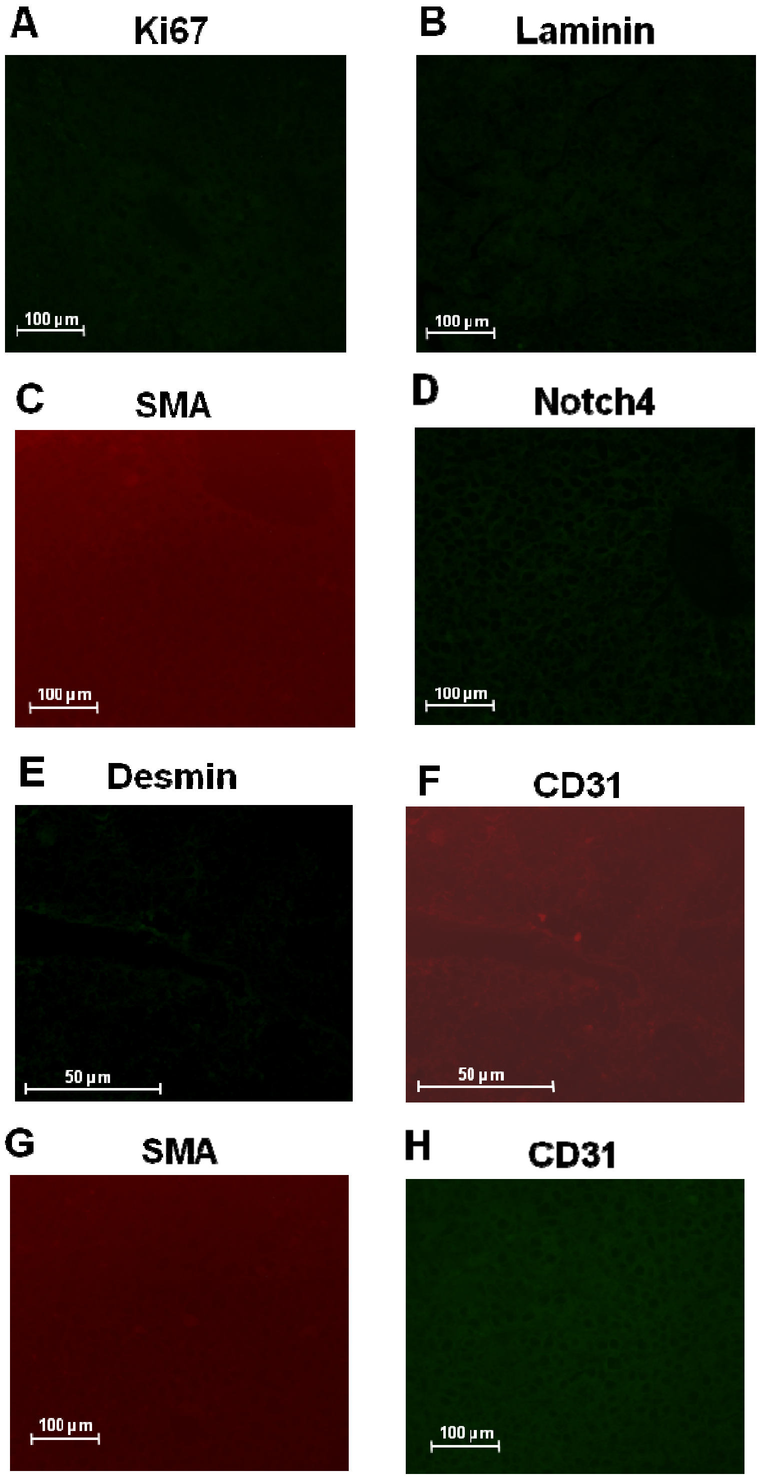

Supplementary Fig. 2

Representative images of negative controls. The primary antibody was omitted, or incubated with an excess of blocking peptide as a negative control.
